# Key Components of Interventions for Grandparents Raising Grandchildren: A Systematic Review

**DOI:** 10.1093/geroni/igaa057.1113

**Published:** 2020-12-16

**Authors:** Athena Chung Yin Chan, Timothy Piehler

**Affiliations:** 1 University of Minnesota–Twin Cities, Saint Paul, Minnesota, United States; 2 University of Minnesota – Twin Cities, St. Paul, Minnesota, United States

## Abstract

Increasingly grandparents provide primary or supplementary care for their grandchildren globally. In view of the challenges faced by grandparent caregivers, a number of intervention programs were designed to act as a buffer to the potential negative impacts on their well-beings. A recent meta-analysis (Chan et al., 2019) shows that interventions for grandparent caregivers have small to moderate effect sizes for increases in social support and parenting skills. However, little is known how the multi-component interventions work. The aim of this review is to categorize the key components of intervention programs and determine the direct effect of components on grandparent caregivers’ outcomes (i.e., physical, mental health and well-being). A component-level approach is adopted to disentangle the ‘active ingredients’ of the interventions. Peer-reviewed articles were searched via five electronic database (published before January 2020). A keyword search was performed based on combinations of two groups of terms: (1) grandparent caregivers and (2) intervention, training, and support group. Studies adopting an experimental or pre/post design and reporting quantitative changes in grandparents’ outcomes were included. Studies focusing on the special needs of grandchildren were excluded. Fourteen interventions were identified from over 20 included studies. Preliminary analyses show that different physical and mental outcomes of grandparent caregivers were predicted from key components of education, skills training, relaxation and psychosocial support. With the goal of developing evidence-based interventions for grandparents raising grandchildren, this synthesis offers insight into the mechanisms of an intervention, which informs its generalizability or applicability to a new context.

